# piRNA-8041 is downregulated in human glioblastoma and suppresses tumor growth *in vitro* and *in vivo*

**DOI:** 10.18632/oncotarget.26331

**Published:** 2018-12-28

**Authors:** Daniel I. Jacobs, Qin Qin, Alan Fu, Zeming Chen, Jiangbing Zhou, Yong Zhu

**Affiliations:** ^1^ Department of Environmental Health Sciences, Yale School of Public Health, New Haven, CT, USA; ^2^ Current address: Division of Genetic and Molecular Toxicology, National Center for Toxicological Research, Food and Drug Administration, Jefferson, AR, USA; ^3^ Current Address: Department of Epidemiology, UCLA Fielding School of Public Health, Los Angeles, CA, USA; ^4^ Department of Neurosurgery, Yale School of Medicine, New Haven, CT, USA; ^5^ Department of Biomedical Engineering, Yale University, New Haven, CT, USA

**Keywords:** piRNA, PIWI-interacting RNA, glioma, GBM

## Abstract

PIWI-interacting RNAs (piRNAs) are small non-coding RNAs that partner with PIWI proteins to protect germline tissues from destabilizing transposon activity. While the aberrant expression of PIWI proteins has been linked with poor outcomes for many cancers, less is known about the expression or function of piRNAs in cancer. We performed array-based piRNA expression profiling in seven pairs of normal brain and glioblastoma multiforme (GBM) tissue specimens, and identified expression of ~350 piRNAs in both tissues and a subset with dysregulated expression in GBM. Over-expression of the most down-regulated piRNA in GBM tissue, piR-8041, was found to reduce glioma cell line proliferation, induce cell cycle arrest and apoptosis, and inhibit cell survival pathways. Furthermore, pre-treatment with piR-8041 significantly reduced the volume of intracranial mouse xenograft tumors. Taken together, our study reveals reduced expression in GBM of piR-8041 and other piRNAs with tumor suppressive properties, and suggests that restoration of such piRNAs may be a potential strategy for GBM therapy.

## INTRODUCTION

PIWI-interacting RNAs (piRNAs) are small (mostly 26-32 nt) noncoding RNAs with highly conserved functions in the protection of germline stem cells from transposable element mobilization [1, 2]. Like microRNAs and small interfering RNAs, piRNAs act as guides in sequence-specific gene regulation, yet are far more abundant - over 30,000 piRNAs have been identified in humans, and based on studies in other species it is likely that far more have yet to be found [3–7]. PIWI-piRNA ribonucleoprotein complexes recruit chromatin-remodeling machinery to complementary transposable element targets, where heritable epigenetic modifications are established (via DNA methylation in mammals) [8–10]; we and others have shown that this may also occur at protein-coding genes [11–13]. Recent studies have also suggested that piRNAs may act post-transcriptionally in mRNA silencing [6, 7, 14, 15].

Despite the longstanding notion that activity of the PIWI-piRNA pathway is restricted to the germline, evidence is quickly mounting for roles in somatic tissues, particularly in the context of cancer [16–19]. Aberrant PIWI-family protein expression has been associated with unfavorable prognosis in eleven cancer types, and piRNA expression has been observed in at least eight cancer types (reviewed in [20, 21]). A recent comprehensive analysis of piRNA expression outside of the germline utilizing RNA-seq data from The Cancer Genome Atlas (TCGA) has demonstrated that hundreds of piRNAs are expressed in both normal and malignant tissues from each of eleven anatomical sites (bladder, breast, colon, head/neck, kidney, lung, ovaries, prostate, stomach, thyroid, and uterus), and that piRNA expression programs are dysregulated in a clinically relevant, tumor type-specific manner [22].

While some progress has been made in documenting piRNA expression in cancer tissue, few studies have elucidated the functional implications of such aberrations in tumorigenesis. Furthermore, no study has examined the role of piRNA expression in glioma, the most common adult primary malignant brain tumor [23], despite reports that some piRNAs are expressed in the mammalian central nervous system [24] and that PIWI-family protein PIWIL1 is associated with glioma tumor growth and prognosis [25, 26]. Here we report results of genome-wide piRNA expression profiling and functional analyses in glioblastoma multiforme (GBM), the most aggressive subtype of glioma.

## RESULTS

### Differentially expressed piRNAs in GBM tissue specimens

**Figure 1:**
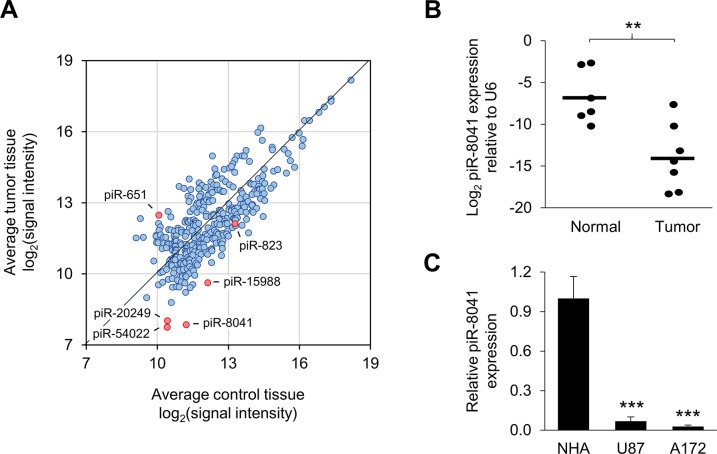
piRNA expression profiling results and confirmation of piR-8041 underexpression in GBM relative to normal brain tissue **(A)** Results of array-based piRNA expression profiling in GBM relative to normal pooled tissue specimens. piRNAs with detectable expression levels are plotted according to average log_2_(signal intensity) in each tissue type. piR-8041 and three additional piRNAs examined in subsequent cell proliferation analyses are labeled (piR-20249, piR-54022, piR-15988), as well as two piRNAs previously shown to be dysregulated in cancer (piR-651, piR-823). **(B)** Validation of piR-8041 expression levels in individual normal vs. tumor tissue specimens by qPCR. Data are presented as log_2_(piR-8041 expression level) relative to small RNA U6 expression; lines denote mean expression level by tissue type. **(C)** Measurement of piR-8041 expression in normal human astrocytes (NHA) and glioma cell lines U87 and A172 by qPCR. ^**^, *P* < 0.01; ^***^, *P* < 0.001; error bars denote standard deviation of triplicate measurements.

Following array-based piRNA profiling, 353 piRNAs were observed to be expressed in both normal and tumor tissue (Figure 1A). Expression differences of at least two-fold between comparison groups were observed for 145 piRNAs (Supplementary Table 3). Among these differentially expressed piRNAs were two that have been previously found to be dysregulated in cancer, piR-651 and piR-823 [27, 28]. Of particular interest was piR-8041, which was 10.3-fold underexpressed in GBM relative to normal tissue and is a 26-nt piRNA encoded by the 12^th^ exon of protein-coding gene *SAPS2* on chromosome 22. The expression difference observed by array profiling was confirmed in individual samples by qPCR (Figure 1B). In agreement with the observation in clinical specimens, piR-8041 was found to be approximately 15- and 35-fold underexpressed in two GBM cell lines, U87 and A172, respectively, relative to NHA cells (Figure 1C). piR-8041 expression was not detectable by northern blot in these cell lines (data not shown). Two PIWI family proteins, PIWIL3 and PIWIL4, were found to be expressed in these cell lines (Supplementary Figure 1).

### Restored expression of GBM-underexpressed piRNAs reduces GBM cell proliferation

**Figure 2:**
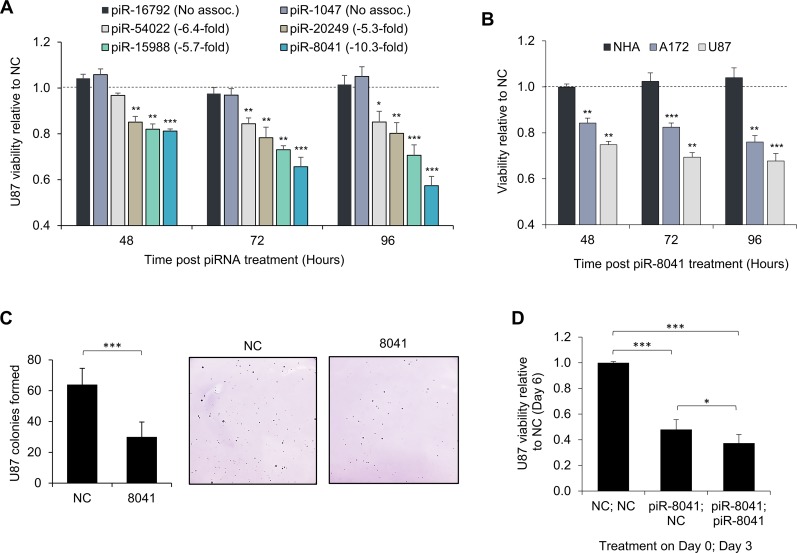
Reduction of GBM cell proliferation by piR-8041 and other GBM-underexpressed piRNAs Growth inhibition was specific to GBM-underexpressed piRNAs and glioma cell lines, piR-8041 reduced long-term colony formation, and growth inhibition was enhanced with a secondary treatment. **(A)** U87 cell proliferation following transfection of piRNAs underexpressed in tumor relative to normal brain tissue (fold-changes noted in figure legend) or piRNAs equivalently expressed in tumor and normal brain tissue (no association). Values denote ratio of color development after MTS exposure of piRNA-treated cells relative to negative control (NC)-treated cells; dotted line represents equivalent cell viability after piRNA or negative control RNA exposure. **(B)** NHA, A172, and U87 cell proliferation following piR-8041 upregulation. Values denote relative viability of piR-8041 vs. NC-treated cells and statistical significance was assessed by the deviation from NC treatment, denoted by the dotted line. **(C)** U87 colonies formed in soft agar 21 days after piR-8041 or NC transfection. Colonies were counted using ImageJ software; representative images are presented. **(D)** U87 cell viability at six days following one (day 0 only) or two (day 0 and day 3) piR-8041 treatments. NS, not significant; ^*^, *P* < 0.05; ^**^, *P* < 0.01; ^***^, *P* < 0.001; error bars denote standard deviation of triplicate experiments for all figures.

To explore the biological significance of our findings, we measured the impact on U87 cell proliferation following exogenous overexpression of piR-8041 and other GBM-underexpressed piRNAs. More than a 30% reduction in cell population viability was observed 96 hours after piR-8041 transfection. We also examined the effect on cell viability of treatment with three other underexpressed piRNAs (piR-54022, piR-20249, and piR-15988), and found that delivery of these piRNAs also reduced viability of U87 cells, though to a lesser degree than piR-8041. Notably, delivery of two piRNAs that were expressed to an equivalent degree between tumor and normal specimens (piR-16792 and piR-1047) did not significantly affect the viability of U87 cell populations (Figure 2A).

Experiments using two other glial cell lines indicated that piR-8041 also inhibited cell proliferation of glioma cell line A172, yet did not affect proliferation of normal human astrocytes (NHA) (Figure 2B). Additionally, soft agar assays were performed to examine the effect of piR-8041 treatment on long-term U87 colony formation. Consistently, piR-8041 treatment significantly reduced the number of colonies formed after three weeks (Figure 2C). We also examined the effect of treating U87 cells a second time with piR-8041 three days after the initial transfection. U87 viability six days after the initial transfection was less than 40% of control-treated cell viability, and statistically significantly less than for cells treated only once. (Figure 2D).

### piR-8041 induces transcriptional changes in cell stress and survival pathways

To characterize the cellular response to piR-8041 treatment, we performed genome-wide transcriptional profiling of piR-8041-exposed U87 cells. The analysis yielded 214 transcripts that were differentially expressed (Supplementary Table 4); 108 were upregulated and 106 were downregulated in piR-8041-treated cells. Gene expression changes measured by qPCR for five top differentially expressed transcripts were found to be consistent with array results (Supplementary Figure 2).

**Figure 3:**
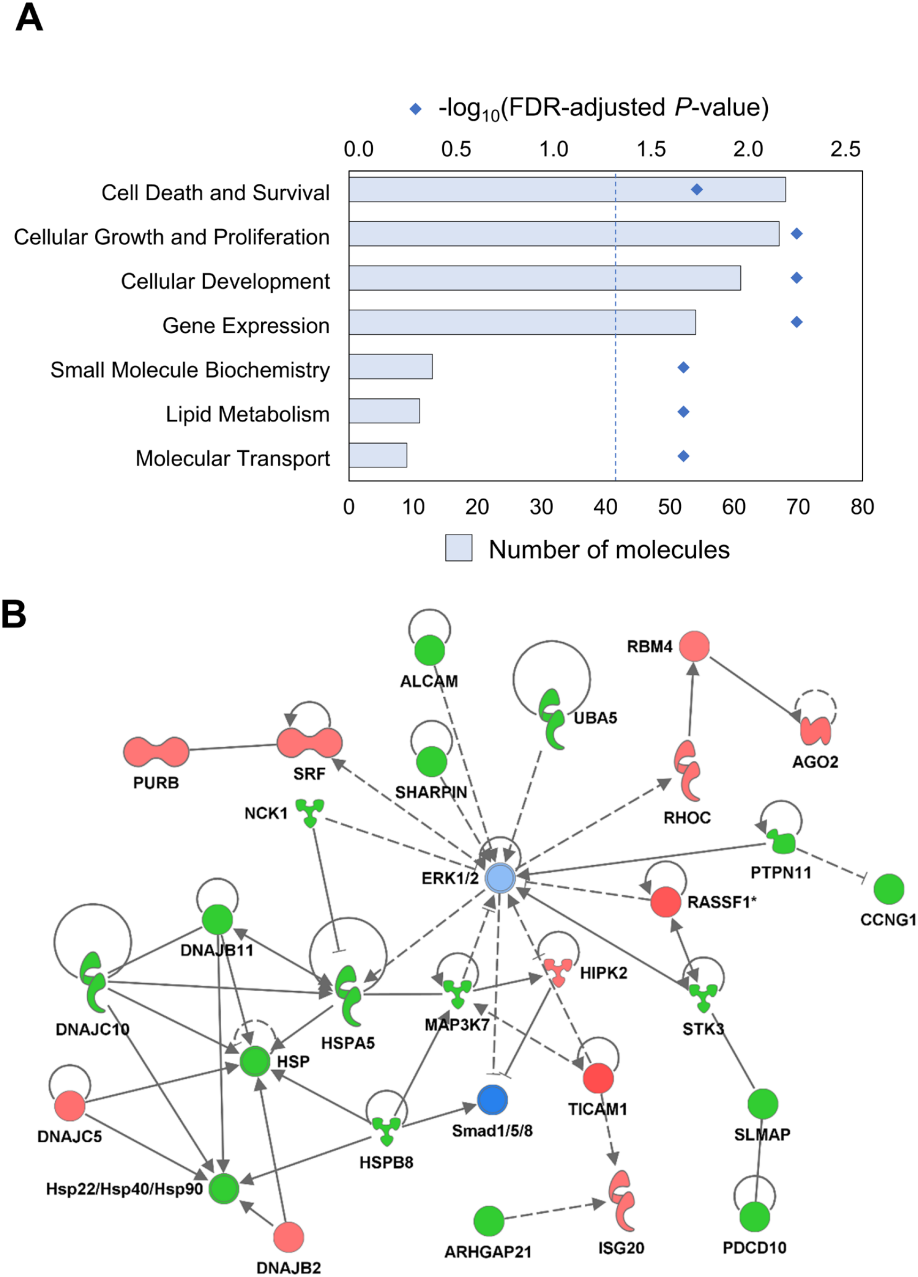
piR-8041 upregulation impacts expression of genes related to cellular survival, stress, and other glioma-relevant functions **(A)** List of biological functions statistically significantly enriched among genes differentially expressed by piR-8041 upregulation in U87 cells. Bars indicate the number of genes impacted with a particular functional annotation; diamonds denote the log-transformed FDR-adjusted *P*-values (dotted line indicates an FDR-adjusted *P*-value of 0.05). **(B)** Illustration of top network of differentially expressed transcripts, related to “decreased cell viability of connective tissue cells” and “decreased synthesis of protein” following piR-8041 treatment of U87 cells. Red and green shading denote transcript over- and under-expression relative to negative control after piR-8041 upregulation, respectively, with color intensity corresponding to degree of change, and blue shading denotes predicted signaling pathway inhibition. Solid lines and dotted lines indicate direct and indirect relationships, respectively.

According to Ingenuity Pathway Analysis, piR-8041-affected transcripts were statistically significantly enriched, after adjustment for multiple comparisons, in seven major functional categories including cell death and survival, cellular growth and proliferation, and cellular development (Figure 3A), and transcriptional changes were predicted to be consistent with “decreased cell viability of connective tissue cells” and “decreased synthesis of protein.” Network analyses indicated that several members of the heat shock protein and related DNAJ Protein chaperone families were suppressed following piR-8041 treatment, as were several transcripts encoding MAPK/ERK signaling pathway proteins, indicating transcriptional impact on cellular stress and survival pathways (Figure 3B).

Additionally, we measured SAPS2 mRNA expression to determine whether piR-8041 acts *in cis* to regulate host gene *SAPS2* and observed a 4-fold reduction following piR-8041 upregulation (Supplementary Figure 3A). However, methylation levels at two CpG islands in proximity to the piR-8041 complementary sequence were found to be unchanged following piR-8041 transfection (Supplementary Figure 3B).

### piR-8041 overexpression induces cell cycle arrest and apoptosis but does not affect invasion or migration of GBM cells

**Figure 4:**
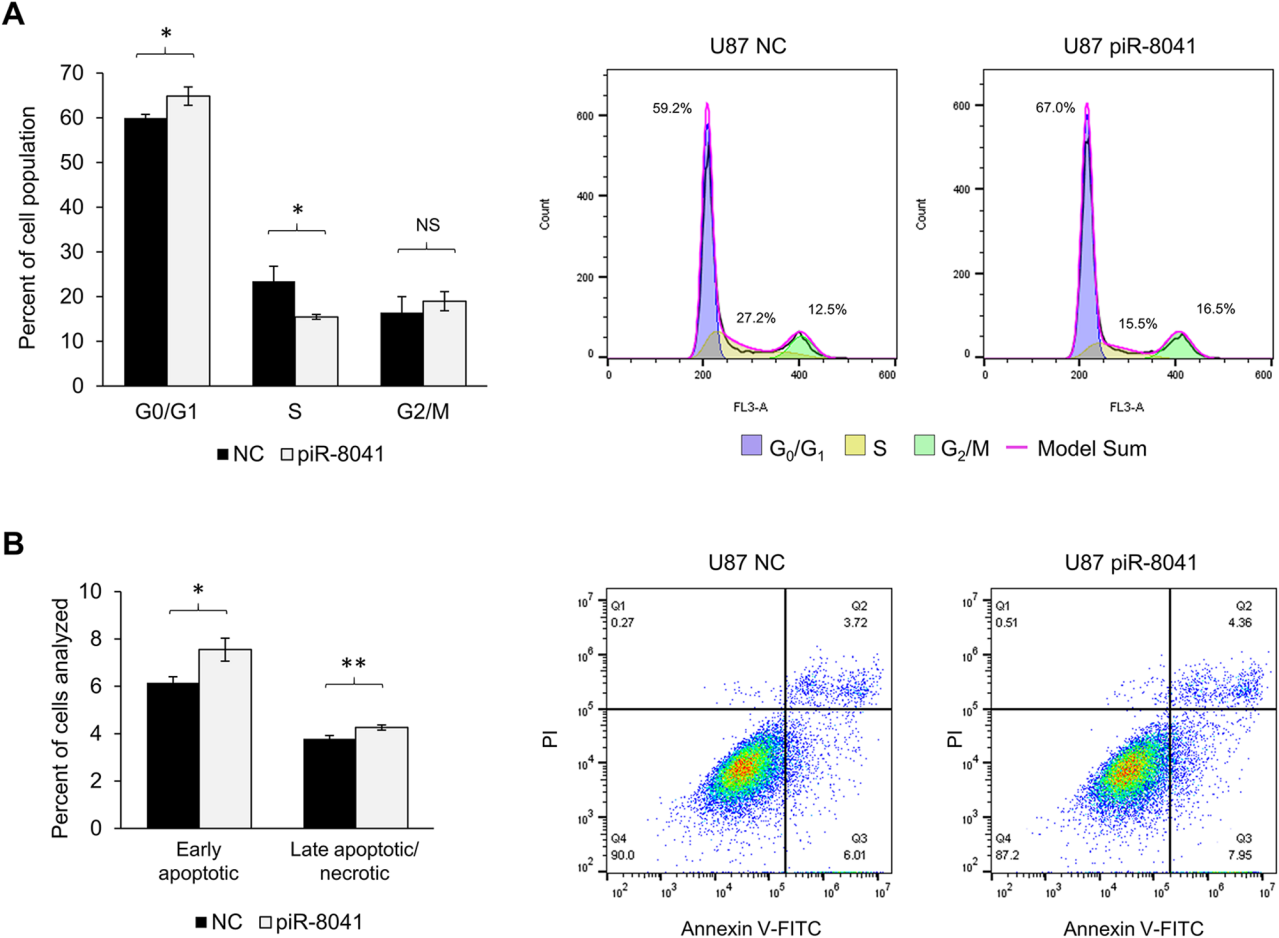
piR-8041 treatment of U87 cells inhibits cell cycle progression by approximately 25% and induces apoptosis **(A)** Cell cycle distribution 48 hours post-piR-8041 or NC-treatment. Cell cycle phases were determined by flow cytometric analysis of DNA content by staining with propidium iodide. Representative cell cycle distributions of NC- and piR-8041-treated U87 cells are shown at right with modeled phase fractions superimposed on DNA content histograms. **(B)** Proportions of U87 cells in early or late apoptosis/necrosis 48 hours post-piR-8041 or NC-treatment. Early apoptotic cells were defined as those stained with Annexin V but excluding PI, late apoptotic/necrotic were cells stained with both probes; representative plots are shown at right. NS, not significant; ^*^, *P* < 0.05; ^**^, *P* < 0.01; error bars denote standard deviation of triplicate experiments.

To investigate the potential anti-proliferative mechanism of piR-8041 treatment, cell cycle and apoptosis assays were performed. DNA content analysis revealed an accumulation of U87 cells at the G_0_/G_1_ checkpoint and a concomitant decrease of the S-phase fraction 48 hours after piR-8041 treatment (Figure 4A). No difference was observed in the proportion of cells in G_2_/M. Additionally, piR-8041 treatment was found to induce statistically significant increases in the proportion of early apoptotic and late apoptotic/necrotic cells (Figure 4B). However, it was observed that U87 and A172 cells were comparably invasive following piR-8041 or control oligo treatment (Supplementary Figure 4A). The migratory ability of GBM cells was also unaffected by piR-8041 treatment, as demonstrated by comparable wound-closure rates on a collagen-coated surface in both U87 and A172 cell lines (Supplementary Figures 4B, 4C).

### *In vivo* tumor growth is temporarily restricted following piR-8041 treatment

**Figure 5:**
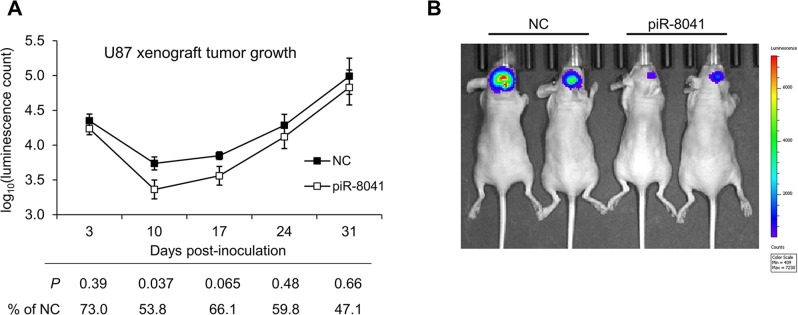
piR-8041 reduces U87 cell growth by nearly 50% 10 days after treatment in an orthotopic xenograft model **(A)** Bioluminescence measurements of luciferase-expressing intracranial tumors at multiple timepoints. Luminescence intensity was measured as a proxy for tumor volume using an IVIS SpectrumCT Imaging System following intravitreal luciferin injection. Statistical significance was assessed by Student's *t*-test between treatment conditions at each time point; associated *P*-values are presented along with average piR-8041-treated tumor intensity as a percentage of control intensity. **(B)** Images of representative mice from each treatment group on day 10 after tumor implantation. Colors correspond to the luminescence scale presented at right, with red and blue coloring representing high and low luminescence intensity, respectively.

To test whether piR-8041 affects glioma cell growth *in vivo*, we implanted luciferase-expressing U87 cells transfected with piR-8041 or negative control RNA intracranially in nude mice. Tumor growth was evaluated in live animals by bioluminescence imaging at 3, 10, 17, 24, and 31 days after implantation. Ten days after implantation, piRNA-treated tumors were approximately 54% of the size and statistically significantly smaller than control-treated tumors, and were 66% of the size and marginally significantly smaller on day 17 (Figure 5A, 5B). While piRNA-treated tumors were still reduced in size during the last two weeks for which measurements were taken, these differences were less pronounced and not statistically significant, suggesting a diminishing impact of transient piR-8041 treatment after approximately 2 weeks.

## DISCUSSION

The PIWI-piRNA pathway has been demonstrated to play a highly conserved regulatory role in transposon suppression in germline stem cells [1, 29]. However, its significance outside of this context remains largely enigmatic, and no previous studies have examined the role of piRNAs in many cancer types, including glioma.

Our array-based piRNA expression profiling results indicated that ~350 piRNAs are expressed in both normal and GBM brain tissue. Interestingly, this number is comparable to the number of piRNAs identified in a panel of other tissues using TCGA small RNA sequencing data [22]. The observation of piRNA expression in normal brain specimens is also consistent with previous work demonstrating a role for PIWI-family proteins and piRNAs in multiple brain functions including memory, neuronal polarization, and stroke [13, 30, 31]. A subset of piRNAs was differentially expressed in tumor tissue, raising the possibility that specific piRNAs may be involved in the tumorigenic process in a similar manner as has been extensively documented for tumor-suppressive and oncogenic microRNAs [32, 33].

Notably, among the identified differentially expressed piRNAs were two, piR-651 and piR-823, that have also been found to be differentially expressed in other cancer types. Data from our study are consistent with previous studies that have reported increased expression of piR-651 in gastric, lung, colon, and breast cancer tissues [28] and decreased expression of piR-823 in gastric cancer tissue [27]. Further functional studies indicated that restoration of piR-823 expression in gastric cancer cell lines inhibited tumor cell growth by 40% *in vitro* and 75% *in vivo* [27] and inhibition of over-expressed piR-651 with antisense oligos resulted in a 30% reduction in tumor growth rate [28]. In light of this prior research, our data suggest that among the piRNAs that are dysregulated in GBM may be a subset that play more universal oncogenic and tumor-suppressive roles across multiple cancer types.

Tumor-suppressive properties of several under-expressed piRNAs in GBM tissue were observed in our study, while delivery of piRNAs with equivalent expression levels between tumor and normal specimens did not affect tumor cell growth. Notably, GBM-underexpressed piR-8041 was shown to have the strongest anti-proliferative effect of the piRNAs tested, yet delivery of piR-8041 did not significantly affect the proliferation of a normal human astrocyte cell line. These results indicate that the observed antitumor effects are not only tumor cell specific but also sensitive to the degrees of underexpression levels, which may be attributable to differences in piRNA targets, the accessibility or abundance of the targets, and/or the expression of required PIWI proteins or associated machinery in different cell types. In an intracranial xenograft mouse model, pre-implantation piR-8041 treatment significantly inhibited tumor growth relative to negative control treatment for approximately 2 weeks, however growth subsequently accelerated. This suggests that repeated treatments will be required to sustain a tumor-suppressive dose of piR-8041, which in clinical practice will depend heavily on the availability of drug delivery vehicles that can cross the blood-brain barrier and deliver an effective dose to the tumor site.

Functional analyses suggested that piR-8041 reduces cell proliferation primarily via induction of cell cycle arrest at the G_1_/S checkpoint, as well as induction of apoptosis in a small proportion of cells. This is consistent with transcriptional profiling data indicating down-regulation of ERK1/2 mitogen-activated protein kinase (MAPK) signaling, the activation of which is required for G_1_/S-phase cell cycle progression [34, 35], as well as observed transcriptional down-regulation of related *MAP3K7*, which encodes a TGF-β-activated kinase whose inhibition has been shown to promote apoptosis in multiple cancer types [36–38]. Furthermore, piR-8041 transcriptionally down-regulated several members of the heat shock protein (HSP) and DNAJ protein families, which facilitate proper protein folding and transport and have been extensively linked to cell stress and tumorigenesis via promotion of cell proliferation and inhibition of death pathways [39–41]; small molecule inhibitors of HSPs (specifically, HSP90) have shown promise as anticancer therapeutics due to the disrupted activity of a large number of HSP-dependent oncoproteins [42, 43].

Among the transcripts impacted by piR-8041 delivery are also several with previously documented roles in glioma. Treatment with piR-8041 led to increased expression of tumor suppressor *RASSF1*, which encodes a tumor suppressor shown to mediate cell cycle arrest at the G_1_/S-phase transition via inhibition of cyclin D1 accumulation [44] and also shown to induce apoptotic cell death [45]. *RASSF1* is frequently epigenetically inactivated in adult glioma via promoter hypermethylation; restoration of *RASSF1* expression sharply restricts glioma cell line colony formation [46]. Additionally, transcripts encoding two genes thought to be potential targets for glioma treatment, *ALCAM*/*CD166* and *SHP-2*, were downregulated upon piR-8041 transfection. Overexpression of glioma stem cell marker *ALCAM*/*CD166* has been shown to promote glioma progression *in vivo*, suggesting clinical value of its targeting for glioma management [47]. *SHP-2* encodes a phosphotyrosine phosphatase that mediates the propagation of Ras/Raf/MAPK growth signaling; inhibition of *SHP-2* has been shown to impair glioma tumorigenesis [48, 49].

It should be noted that piR-8041-mediated transcriptional changes observed may have been either direct or indirect in nature, and that future work will be required to determine the direct targets of piR-8041 and detailed mechanism of action such as its interaction with PIWI proteins. Our finding that piR-8041 host gene *SAPS2* expression is reduced after piR-8041 transfection without an appreciable change in regional DNA methylation suggests that piR-8041 may act in an siRNA-like manner to silence complementary targets, which is consistent with recent studies indicating post-transcriptional mRNA silencing by piRNAs [6, 7, 14, 15]. However, *SAPS2* itself does not have an apparent relevance to tumorigenesis and thus the tumor-suppressive effect of piR-8041 is likely mediated by the targeting of other unknown sequences of imperfect complementarity. We also note that piRNA overexpression experiments were designed to test the cellular impact of the piRNAs in question, although the expression levels achieved were likely higher than what would be seen under normal physiological conditions.

It is also possible that long RNAs with the same specific 3′-ends might also be detected in both array and qPCR assays. However, like microRNAs and other small noncoding RNAs, these long RNA fragments could be the piRNA precursors that eventually contribute to the amount of the mature piRNAs. This initial expression screening helps us generate potential piRNA candidates for hypothesis testing in the following functional analyses. In fact, detections of several piRNAs with altered expressions in this study are consistent with findings from other previous publications that have been described above. Moreover, underexpressed piRNAs significantly reduced viability of GBM cells in a dose-response manner. The antitumor effect of the top piRNA candidate piR-8041 was further confirmed in the following multiple *in vitro* assays and the animal study. As such, we believe that the expression screening can successfully identify functional piRNAs in GBM that warrants further mechanistic investigations in the future.

Taken together, the functionally-relevant dysregulation of piRNA expression in GBM identified in this study sheds new light on the biology of gliomagenesis and suggests that restoration of down-regulated piRNAs may be a viable therapeutic strategy in a manner analogous to “microRNA replacement therapy” of down-regulated tumor-suppressive microRNAs, which is in current clinical testing [50]. Additional mechanistic work and piRNA expression profiling in an expanded set of specimens is warranted in order to pinpoint additional GBM-relevant piRNAs and to more fully understand this novel aspect of GBM biology.

## MATERIALS AND METHODS

### Study specimens and processing

Formalin-fixed paraffin-embedded (FFPE) primary GBM (n=7) and normal brain specimens (n=7; specimens collected post-mortem or from resection for epileptic management), matched by age, race, and gender, were purchased from the Cooperative Human Tissue Network (Supplementary Table 1). Subjects providing tumor specimens had not undergone radio- or chemotherapy at the time of resection. RNA was isolated from sections corresponding to approximately 8-10 mg of tissue from each specimen using the AllPrep DNA/RNA FFPE Kit (QIAGEN). The study was approved by the institutional review board (IRB) of Yale University (HIC Protocol #: 1212011202).

### piRNA expression profiling

Total RNA was pooled in equal proportions by tissue type (tumor and normal) and samples were submitted to ArrayStar facilities for piRNA expression profiling in duplicate using the ArrayStar Human 4×44K piRNA Expression Array, which includes probes for 23,677 mature human piRNAs. Data were quantile normalized with Agilent GeneSpring GX 12.1 software and have been deposited to the Gene Expression Omnibus repository (GSE79438). piRNAs with signal intensity >2,000 were considered to be expressed and differences between sample types were calculated to assess biologically significant changes.

### Cell lines and reagents

Glioma cell lines U87 and A172, purchased from ATCC, and immortalized normal human astrocytes (NHA), purchased from the University of California, San Francisco Tissue Core, were maintained in EMEM (U87) or DMEM (A172, NHA) supplemented with 10% FBS. All ATCC cell lines are tested for contaminants and authenticated prior to shipment; cells were not re-authenticated as they were passaged in our laboratory for fewer than 6 months after resuscitation. piRNA mimics were purchased from IDT (Supplementary Table 2), andsingle-stranded non-targeting RNA sequences of similar size were used as negative control. For *in vitro* assays, cells were reverse transfected according to the manufacturer's instructions using LipofectAMINE RNAiMAX transfection reagent (Invitrogen); transfection efficiency was confirmed using siGLO fluorescent transfection control oligo (GE Dharmacon) (Supplementary Figure 5).

### Protein extraction and western blotting

Standard immunoblotting technique was used. Briefly, total protein lysate was collected from U87, A172, and NHA cells using RIPA buffer (Santa Cruz Biotechnology) and 30 ug protein was run on a 10% NuPAGE Bis-Tris gel (Life Technologies) and transferred to PVDF membranes. Membranes were incubated overnight at 4°C with the following primary antibodies: PIWIL1 (Abcam 12337, PIWIL2 (Abcam 36764), PIWIL3 (Novus Biologicals 31855), PIWIL4 (Abcam 111714) or β-actin (Santa Cruz Biotechnology 47778).

### Confirmation of piRNA expression

piR-8041 expression was quantified in individual patient specimens and U87, A172, and NHA total RNA by qPCR with locked nucleic acid (LNA) probes for enhanced specificity and sensitivity. Briefly, RNA was reverse transcribed using an Exiqon Universal cDNA Synthesis Kit and targets were amplified in triplicate using custom piR-8041 primers with the ExiLENT SYBR Green PCR Kit (Exiqon) with normalization to small nuclear RNA U6 expression. During cDNA synthesis, a universal tag was added to the mature piRNA and a reverse PCR primer spanning the piRNA-tag junction was used, which ensured amplification specificity of the piRNA of interest as opposed to any longer matching sequence. This technology from Exiqon is explicitly designed for specific amplification of small RNA species. Northern blotting was also performed using standard technique.

### Cell viability and soft agar assays

For cell viability, cells were reverse transfected with piRNA or negative control oligos and color development was evaluated one hour after addition of MTS (Promega) using a microplate spectrophotometer. For soft agar assays, cells were reverse transfected with piRNA or negative control oligos. After 24 hours, cells were re-suspended in warmed culture medium with 0.36% agar and seeded in 60 mm dishes above a base layer of 0.75% agar. Colonies were stained with 0.04% crystal violet-2% ethanol in PBS after three weeks and counted using ImageJ v1.48 software. Experiments were performed in triplicate and differences of viability and colony number were analyzed using a Student's *t*-test.

### Genome-wide transcriptome profiling

RNA profiling of piR-8041- or control RNA-transfected U87 cells, 24 hours post-transfection, was performed on the Illumina HumanHT-12 v4 Expression BeadChip platform in biological duplicate. Genes with expression differences ≥ |1.2|-fold and beyond a significance threshold of FDR-adjusted *P* = 0.05 were considered to be differentially expressed, and 5 genes were selected for expression validation by qPCR with input normalization to *GAPDH* (primers shown in Supplementary Table 2). Ingenuity Pathway Analysis software was used to perform network analyses and identify affected functional pathways using a Fisher's exact test for enrichment of genes with a specific functional annotation. Expression array data have been deposited to the Gene Expression Omnibus repository (GSE79438).

### Cell cycle and apoptosis assays

For cell cycle analyses, cells were fixed in 70% ethanol, washed, and incubated with RNase A (100 μg/ml) followed by propidium iodide (PI) (40 μg/ml) in PBS. Cells were then analyzed on a BD FACSCalibur flow cytometer, and G_0_/G_1_, S, and G_2_/M fractions were determined using FlowJo software v10. For apoptosis assays, cells were prepared using the Dead Cell Apoptosis Kit with Annexin V FITC and PI (ThermoFisher Scientific) according to the manufacturer's instructions. Cells were analyzed for Annexin V staining and PI exclusion using a BD Accuri C6 flow cytometer and accompanying software. Differences in apoptotic and cell cycle distributions were analyzed by Student's *t*-test for triplicate experiments.

### Cell invasion and migration assays

For cell invasion assays, piR-8041 or negative control-transfected cells were transferred to the top chamber of a BioCoat Matrigel Invasion Chamber (BD Biosciences) in serum-free media 48-hours post-transfection. After 24 hours, invading cells were fixed and stained, then counted using an Olympus BX51 microscope with a QImaging CCD digital camera. For cell migration assays, cells were reverse transfected in collagen-coated 6-well plates. At 48 hours post-transfection, a scratch was made using a sterile pipette tip and photographs were taken in three separate fields for each condition at baseline, 6 hours, and 12 hours post-scratch. The gap width was measured to calculate the closure percentage relative to baseline. Experiments were performed in triplicate. A two-sided Student's *t*-test was used to compare mean counts of invaded cells and mean closure percentages between piRNA-treated and control conditions in cell invasion and migration assays, respectively.

### piRNA-induced host gene expression and methylation

Gene expression and DNA methylation of piR-8041 host gene *SAPS2* were measured 48 hours after U87 transfection with piR-8041 or negative control. Gene expression was measured by qPCR in triplicate with normalization to *GAPDH*. DNA methylation was evaluated by methylation-specific-PCR in the *SAPS2* exon to which piR-8041 maps as well as an intronic CpG island approximately 1 kb downstream. All primers are shown in Supplementary Table 2.

### *In vivo* tumorigenicity assay

Nude mice (n=9 per group) were anesthetized and placed in a stereotactic frame, and an incision was made and a hole drilled above the right striatum. Approximately 5×10^4^ luciferase-expressing U87 cells suspended in phosphate buffered saline, transfected 24 hours prior to surgery with piR-8041 or control RNA, were injected into the brain and the hole was closed with bone wax and the scalp closed with surgical staples. Following surgery, tumors were imaged using an IVIS SpectrumCT Imaging System (PerkinElmer) following intravitreal luciferin injection, and bioluminescent intensity was measured and compared at each time point by Student's *t*-test. Mice were sacrificed when ethically necessary due to clinical symptoms or substantial loss in body weight. Animal work was approved by the Yale University Institutional Animal Care and Use Committee (IACUC) (Protocol No: 2013-11550).

## SUPPLEMENTARY MATERIALS FIGURES AND TABLES






