# Supplementary material for: piRNA-8041 is downregulated in human glioblastoma and suppresses tumor growth in vitro and in vivo
Source: Oncotarget. 2018 Dec 28;9(102):37616–26. doi: 10.18632/oncotarget.26331 (PMC6340885; doi:10.18632/oncotarget.26331)
Supplement: Supplementary file 1 [file oncotarget-09-37616-s001.pdf]

piRNA-8041 is downregulated in human glioblastoma and suppresses tumor growth *in vitro* and *in vivo*

SUPPLEMENTARY MATERIALS

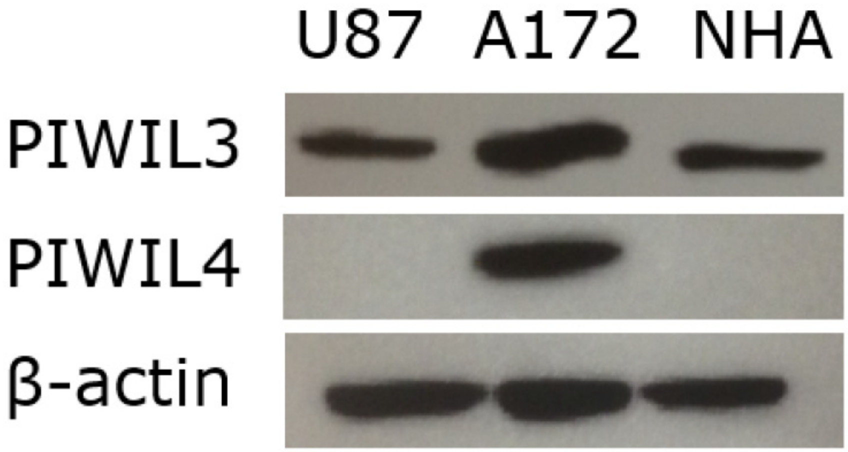

Supplementary Figure 1: Measurement of PIWI family protein expression in U87, A172, and NHA cell lines. Western blotting was performed to measure expression of PIWI family proteins PIWIL1, PIWIL2, PIWIL3, and PIWIL4 with β-actin as loading control. Expression of PIWIL1 and PIWIL2 was not observed in these cell lines.

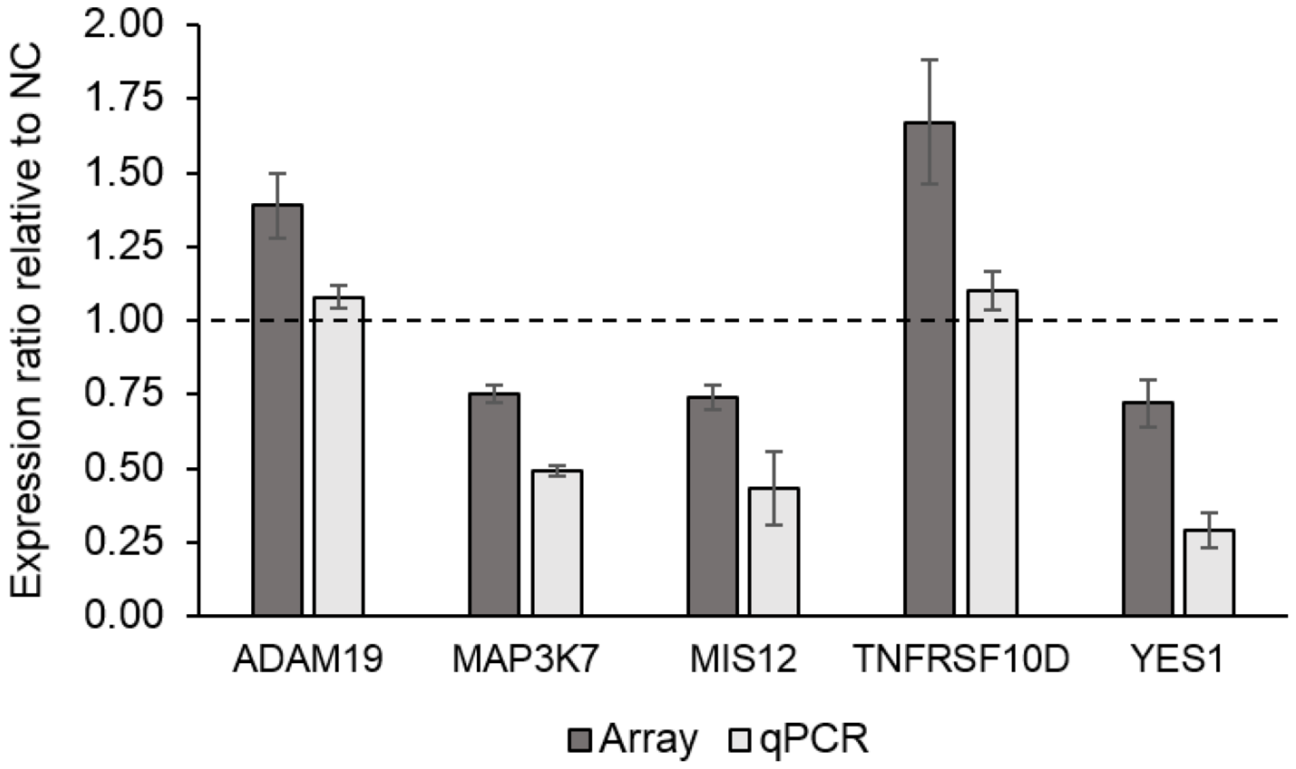

Supplementary Figure 2: Expression array validation results for piR-8041 treatment. Five genes were selected for expression array confirmation by qPCR. Differences are presented as expression ratios of piR-8041-treated U87 cells relative to NC-treated U87 cells; *GAPDH* expression was measured to normalize expression values. qPCR-based expression results were consistent with array findings. Error bars denote standard deviation of three replicates; primers are presented in Supplementary Table 2.

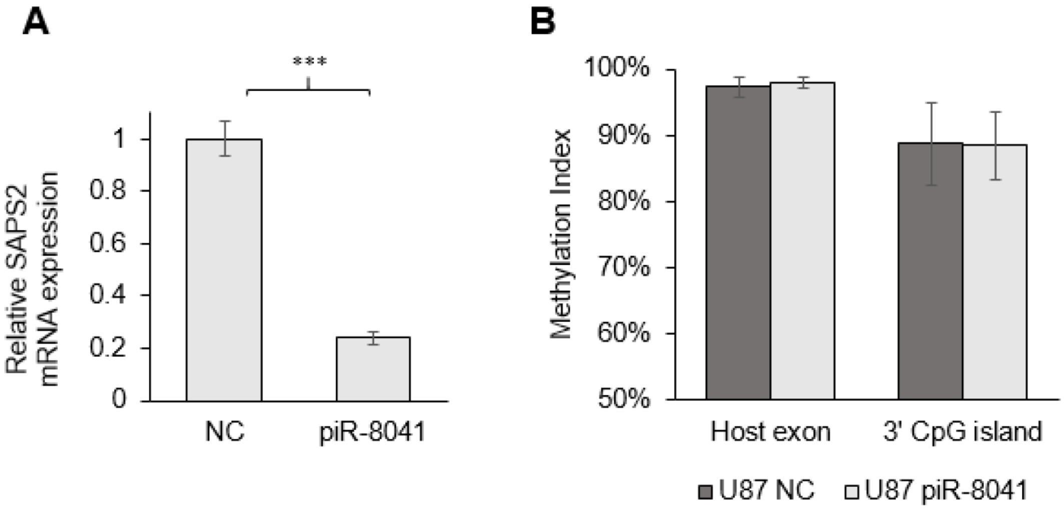

Supplementary Figure 3: Measurement of SAPS2 mRNA expression and methylation change following piR-8041 transfection in U87 cells. **(A)** SAPS2 mRNA expression was measured by qPCR 48 hours after piR-8041 transfection. Expression levels were compared after normalization to HPRT. **(B)** Methylation levels of CpG sites in the piR-8041 host exon and a nearby CpG island were measured by MS-PCR 48 hours post piR-8041 transfection and bisulfite conversion of isolated DNA. ***, $P < 0.001$; error bars denote standard deviation of triplicate experiments.

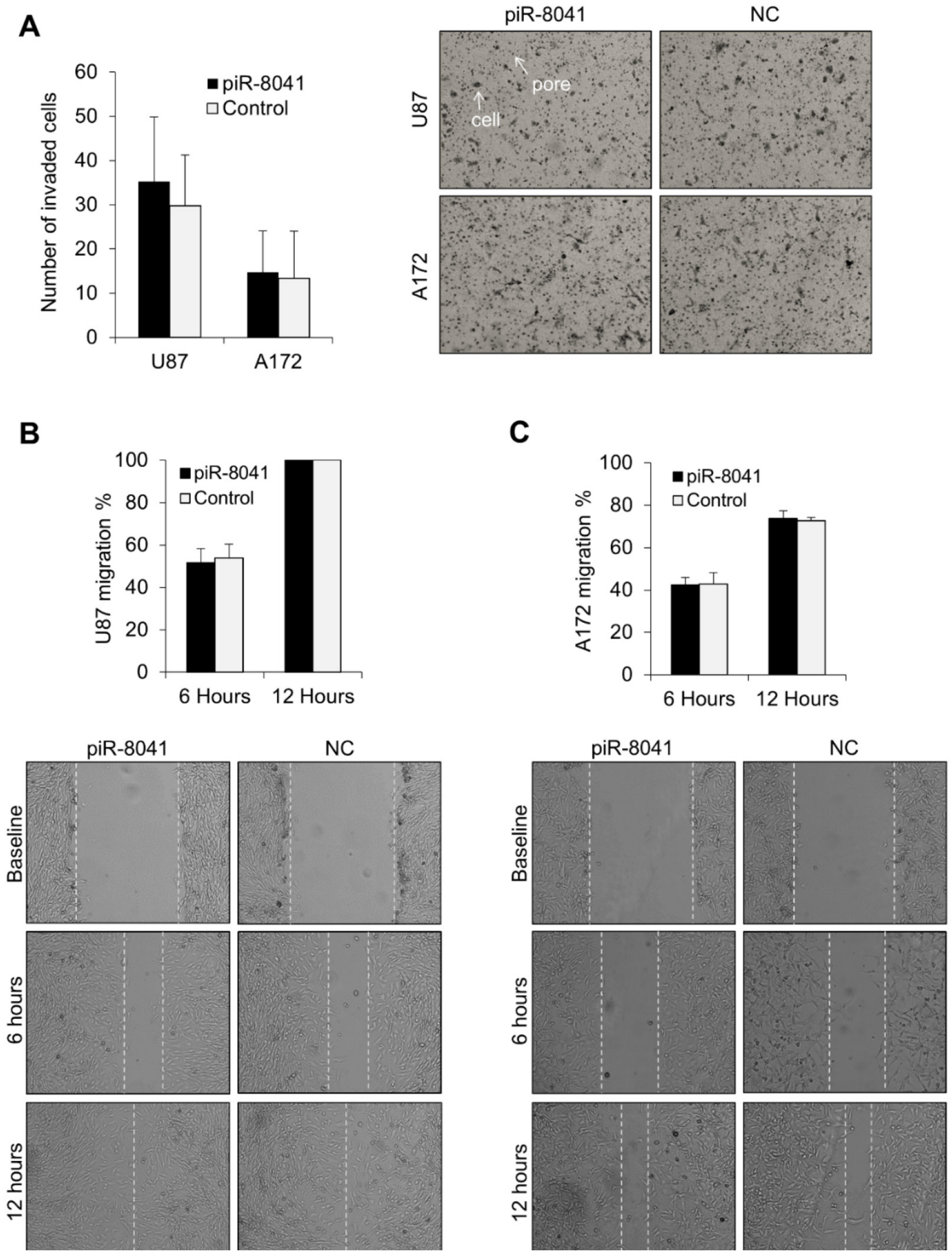

Supplementary Figure 4: piR-8041 does not affect glioma cell invasion or migration. **(A)** U87 and A172 invasion through a Matrigel invasion chamber. piR-8041 or control-treated cells invading through the gel matrix and porous membrane were fixed, stained, and counted after 24 hours. Representative images are shown at right; arrows indicate examples of stained cells and membrane pores. **(B, C)** U87 and A172 migration as evaluated by a scratch assay. piR-8041 or negative control-treated cell monolayers were scratched, and migration percentage was evaluated 6 and 12 hours later as the percentage closure relative to baseline. Dotted lines are used to illustrate the average migration front at each time point. Error bars denote standard deviation of replicate measurements.

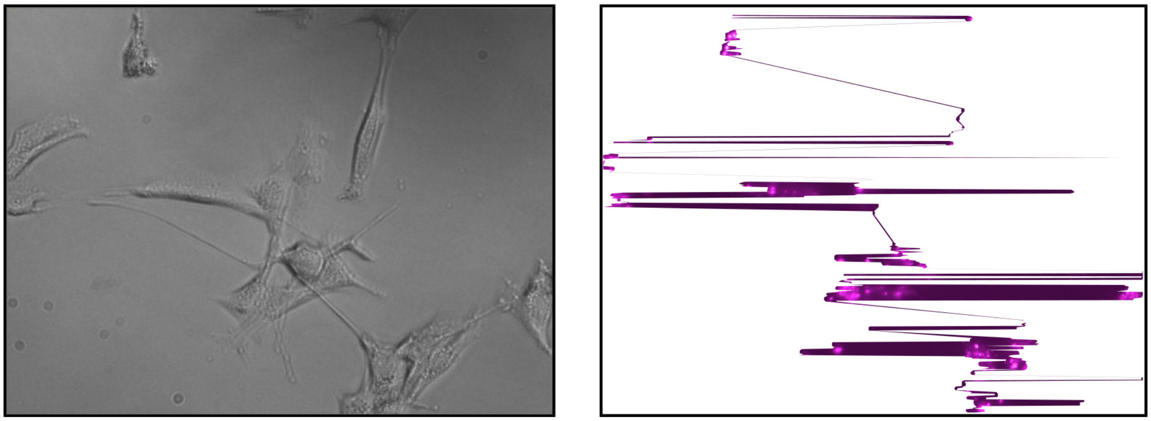

Supplementary Figure 5: Confirmation of transfection efficiency in U87 using siGLO fluorescent transfection control oligo.

Supplementary Table 1: Characteristics of subjects providing specimens for piRNA expression profiling

Characteristic	Diseased (n=7)	Normal (n=7)
Sex[1]		
Male	6 (85.7)	5 (71.4)
Female	1 (14.3)	2 (28.6)
Age[2]	51.0 ± 16.9 (47)	53.6 ± 20.3 (48)
Ethnicity[1]		
Caucasian	6 (85.7)	6 (85.7)
Unknown	1 (14.3)	1 (14.3)

[1]Values are presented as frequency (column percent)
[2]Values are presented as mean ± standard deviation (median)

Supplementary Table 2: Primers used for qPCR and MS-PCR reactions and piRNA mimics used for *in vitro* functional analyses

Amplification target[1]	Forward primer	Reverse primer
TNFRSF10D	5'-TTCTCCACAGTAGCCCAGTC-3'	5'-GGCCATCCCCTCCTAAAACT-3'
ADAM19	5'-CACTTGCCCCAAAGTTTCCA-3'	5'-GCCAGACATGCTTCTTCAGG-3'
YES1	5'-AGGCAGGAAAAGTTGATGGT-3'	5'-AGACACTGAAGACGAAAAGCTG-3'
MIS12	5'-TGTGCCAAAGTCACAGGTTTT-3'	5'-TGCCATCCAGCTTCTTCAGA-3'
MAP3K7	5'-ACAGAGAAAGCAAGAACTAGTTG-3'	5'-TTGTCGTTTCTGCTGCTGAC-3'
GAPDH	5'-GAAGGTGAAGGTCGGAGTCAAC-3'	5'-CAGAGTTAAAAGCAGCCCTGGT-3'
SAPS2	5'-GCCCAAGAAGAAAGCGATCC-3'	5'-GTAAGTCCATCGTGTTGAGCC-3'
SAPS2 Exon M	5'-GTTTTTTGTGTTTGTAGAAGAAAGC-3'	5'-ACTTACCAACAATAAATCCATCGTA-3'
SAPS2 Exon U	5'-TTTTTTGTGTTTGTAGAAGAAAGTGA-3'	5'-ACTTACCAACAATAAATCCATCATA-3'
SAPS2 CpG M	5'-TTTAGTTTTAGAATTGGGTTCGAAG-3'	5'-AACAATACCCAAAACTCATACACG-3'
SAPS2 CPG U	5'-TAGTTTTAGAATTGGGTTTGAAG-3'	5'-AATACCCAAAACTCATACACACTAA-3'
**piRNA mimic**	**Sequence**	
piR-8041	5'-UGAGGCGGGCGCCAUGCAGACGGGCA-3'	
piR-16792	5'-CCUCCCAAAGUGCUGGGAUUACAGGCGUGAG-3'	
piR-54022	5'-UGGACGGACGGAUGGCCAGAUGAAUCAAAACU-3'	
piR-1047	5'-AGGGUAACUAUCAUCAUGUGUCUGGAGGUC-3'	
piR-20249	5'-GGAUAUGGAAAUGAGAGGACGGACAAGC-3'	
piR-15988	5'-UGUGGACUGUCUCAGGAGGCAGAGGGGCACC-3'	

[1]M=methylated; U=unmethylated

Supplementary Table 3: piRNAs differentially expressed at least two-fold in malignant relative to normal brain tissue from piRNA expression profiling

See Supplementary File 1

Supplementary Table 4: Differentially expressed transcripts 24-hours post piR-8041 or NC transfection in U87 (FDR-adjusted $P < 0.05$; fold-change ≥ |1.20|) by fold-change

See Supplementary File 2
